# E-Cadherin Marks a Subset of Inflammatory Dendritic Cells that Promote T Cell-Mediated Colitis

**DOI:** 10.1016/j.immuni.2010.03.017

**Published:** 2010-04-23

**Authors:** Karima R.R. Siddiqui, Sophie Laffont, Fiona Powrie

**Affiliations:** 1 Sir William Dunn School of Pathology, University of Oxford, OX1 3RE Oxford, UK; 2 Translational Gastroenterology Unit, Nuffield Department of Clinical Medicine, Experimental Medicine Division, John Radcliffe Hospital, OX3 9DU Oxford, UK

**Keywords:** MOLIMMUNO, CELLIMMUNO

## Abstract

Dendritic cells (DCs) play a pivotal role in controlling the balance between tolerance and immunity in the intestine. Gut conditioned CD103^+^ DCs promote regulatory T (Treg) cell responses; however, little is known about DCs that drive inflammation in the intestine. Here, we show that monocyte-derived inflammatory DCs that express E-cadherin, the receptor for CD103, promote intestinal inflammation. E-cadherin^+^ DCs accumulated in the inflamed mesenteric lymph nodes and colon, had high expression of toll-like receptors, and produced colitogenic cytokines, such as IL-6 and IL-23, after activation. Importantly, adoptive transfer of E-cadherin^+^ DCs into T cell-restored immunodeficient hosts increased Th17 cell responses in the intestine and led to exacerbation of colitis. These results identify a monocyte-derived inflammatory DC subset that is associated with the pathogenesis of intestinal inflammation, providing a therapeutic target for the treatment of inflammatory bowel disease.

## Highlights

► E-cadherin^+^ DCs accumulate in the inflamed MLN and colon ► E-cadherin^+^ DCs derive mainly from Gr1^+^ inflammatory monocytes ► E-cadherin^+^ DCs produce high amounts of inflammatory cytokines and chemokines ► E-cadherin^+^ DCs exacerbate colitis

## Introduction

The intestine is a unique microenvironment that must constantly maintain a delicate balance between effector and tolerogenic T cell responses because inadequate protective immunity may result in opportunistic infections, whereas aberrant reactions against the commensal flora may trigger chronic intestinal pathologies, such as inflammatory bowel disease (IBD) (Xavier and Podolsky, 2007).

Dendritic cells (DCs) are specialized antigen-presenting cells that play a pivotal role in shaping the host's immune response (Steinman et al., 2003). Several types of DCs have been described according to their origin, location, receptor expression, and functional characteristics. Secondary lymphoid organs contain both conventional resident and migratory DCs, but the relative functional contribution of these subsets in controlling tissue immunity is poorly understood (Geissmann et al., 2008). In addition, monocyte-derived DCs accumulate in infected tissue sites where they promote protective innate and adaptive responses (León et al., 2007; Nakano et al., 2009; Salazar-Gonzalez et al., 2006; Serbina et al., 2003).

Alterations in intestinal DCs are thought to contribute to the effector pathways that lead to IBD. Both in human Crohn's patients and in murine colitis models, pathology is associated with the local accumulation of activated DCs (Hart et al., 2005; Uhlig et al., 2006b). In colitic mice, mesenteric lymph node (MLN) DCs express elevated amounts of CD40 and CD134L, and blockade of either the CD40-CD40L (Uhlig et al., 2006b) or the CD134-CD134L (Malmström et al., 2001) pathway inhibits colitis, illustrating that DCs promote colitogenic responses. However, which particular DC subsets contribute to chronic T cell-mediated colitis is not known.

Precisely how DCs within the same tissue can drive both tolerance and immunity is poorly understood. One hypothesis is that these distinct functions are performed by different DC subsets, which may be segregated both temporally and spatially within the tissue in order to maintain both homeostasis and rapid protective responses upon demand. We and others have previously identified a functionally distinct population of DCs that are enriched within the gut-associated lymphoid tissue (GALT) and which express the integrin α chain CD103 (α_E_) (Johansson-Lindbom et al., 2005). In earlier studies, we found that regulatory T (Treg) cells were unable to suppress colitis in hosts that lacked CD103, indicating a functional role for CD103^+^ DCs in maintaining gut homeostasis (Annacker et al., 2005). Furthermore CD103^+^, but not CD103^−^, MLN DCs were able to promote the differentiation of Treg cells, suggesting a role for this population in the support of both conventional and antigen-induced Treg cells in the GALT (Coombes et al., 2007; Sun et al., 2007). Recent elegant studies have shown that the two major populations of CD11c^hi^ DCs in the intestine have distinct origins. Thus, CD103^+^CX_3_CR1^−^ DCs arise from the common DC progenitor (CDP) via a pre-DC intermediate, whereas CD103^−^CX_3_CR1^+^ DCs develop from Gr1^hi^ monocytes (Bogunovic et al., 2009; Varol et al., 2009). Developmental differences may therefore account for the distinct functional features of CD103^+^ and CD103^−^ DC subsets in the GALT.

To date, only one ligand of CD103 has been identified, which is E-cadherin (Cepek et al., 1994). E-cadherin is a single-span transmembrane glycoprotein that, as well as binding to CD103, also undergoes homotypic interactions that form the core of intercellular tight junctions (Takeichi, 1991). Consequently, E-cadherin has a central role in the maintenance of tissue architecture and early embryonic development (Larue et al., 1994). Although it is widely acknowledged that E-cadherin is a marker of nonhematopoietic intestinal epithelial cells (IECs), E-cadherin is also expressed by bone marrow-derived DCs (BM-DCs) (Jiang et al., 2007), keratinocytes (Tang et al., 1993), and Langerhans cells (LCs) (Riedl et al., 2000). Recently, it has been demonstrated that E-cadherin-stimulated DCs promote Treg cells and that the E-cadherin-β-catenin signaling pathway may be involved in the acquisition of an immunosuppressive DC phenotype in vitro (Jiang et al., 2007). The distribution and function of E-cadherin^+^ DCs in vivo is not known.

Here, we have identified and characterized a population of E-cadherin^+^ DCs that accumulates within the intestine and GALT during T cell-mediated colitis. Our results suggest that E-cadherin^+^ DCs represent an inflammatory monocyte-derived DC population that can promote chronic pathological inflammatory responses in the gut.

## Results

### E-Cadherin Is Expressed by a Minor Population of CD11c^hi^ DCs in Normal Mice

E-cadherin has been shown to be expressed primarily by nonhematopoietic IECs, but there are exceptions, including LCs and BM-DCs (Jiang et al., 2007; Riedl et al., 2000). We first analyzed whether E-cadherin was expressed by DCs in secondary lymphoid tissue. Tissues were harvested, and CD11c-enriched cell suspensions were prepared and subject to flow cytometry analysis. To focus on DCs, cells were gated on the high-forward and side-scatter cell population, which removed the majority of CD11c^−^ cells, and 7-AAD^+^ dead cells were excluded. E-cadherin expression among the CD11c^hi^MHC class II^hi^ population was examined. This gating strategy was applied to all experiments unless otherwise stated (Figure S1A available online). We found that a minor population of CD11c^hi^ cells in the MLN, axillary LN (ALN), and spleen of normal mice expressed E-cadherin (Figure S1B). E-cadherin^+^CD11c^hi^ cells from the MLN expressed similarly high amounts of MHC class II, and the costimulatory molecules CD80, CD86, and CD40 as the reciprocal E-cadherin^−^CD11c^hi^ population, and, hence, displayed the phenotypic characteristics of DCs (Figure S1C). E-cadherin^+^CD11c^hi^ cells did not express Gr1, and they were heterogeneous for CD8α and CD11b, indicative of a mixed DC population. The majority of E-cadherin^+^ DCs at steady state also expressed CD103 (Figure S1D).

### E-Cadherin^+^ DCs Accumulate during T Cell-Mediated, but Not Innate, Colitis

**Figure 1:**
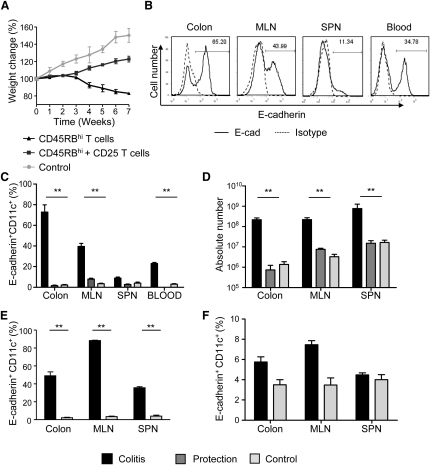
E-Cadherin^+^ DCs Accumulate during T Cell-Mediated Colitis (A–D) BALB/c *Rag2*^−/−^ mice were reconstituted with 4 × 10^5^ CD4^+^CD45RB^hi^ T cells and were sacrificed when displaying clinical signs of colitis. Transfer of 2 × 10^5^ CD4^+^CD25^+^ T cells in combination with the CD4^+^CD45RB^hi^ T cells suppressed both weight loss and disease (termed protected). (A) Weight as a percentage of the initial weight at day 0. Triangle, colitic mice; square, protected mice; circle, non-T cell reconstituted *Rag2*^−/−^ mice. (B) Colon, MLN, spleen, and blood cell preparations from colitic mice were gated on CD11c^+^ DCs, and expression of E-cadherin was assessed. Representative staining is shown. Solid black line, E-cadherin; dashed line, E-cadherin isotype control. (C) Mean frequency and (D) cell number of CD11c^+^ cells expressing E-cadherin isolated from colitic colon, MLN, spleen, and blood. (E) B6 *Rag1*^−/−^ mice were injected with either 200 μg α-CD40 mAb or PBS, and after 7 days, colon, MLN, and spleen cell preparations were prepared. Mean frequency of CD11c^hi^ cells expressing E-cadherin. (F) 129SvEv *Rag2*^−/−^ mice were infected with *H. hepaticus*, and 8 weeks later, colon, MLN, and spleen cell preparations were prepared. Mean frequency of CD11c^+^ cells expressing E-cadherin. Black bars, inflamed; open bars, control; dark gray bars, protected. Error bars represent SD. In all cases, the experiments were repeated more than two times, with each experimental group containing more than five individual mice. See also Figure S1.

We have previously shown that intestinal inflammation is characterized by an accumulation of activated DCs within the GALT (Malmström et al., 2001). However, very little is known about the role of specific DC subsets in chronic T cell-mediated intestinal inflammation. To further investigate this, we used the T cell transfer model of colitis, where transfer of CD4^+^CD45RB^hi^ T cells to immunodeficient hosts leads to mucosal and systemic inflammation. In this model, colitis was associated with a more than 10-fold increase in the frequency of E-cadherin^+^ DCs in the colon and MLN compared to unreconstituted *Rag2*^−/−^ control mice (Figures 1B and 1C), and a more than 3-fold increase in the frequency of E-cadherin^+^ DCs compared to WT mice (Figure S2A). Changes in frequency did not reflect the loss of another subset because there were also large increases in the total number of E-cadherin^+^ DCs in the inflamed MLN and colon (Figure 1D). E-cadherin^+^ DCs in the MLN and colon may derive from a blood precursor because E-cadherin^+^CD11c^int^ cells were present in the blood of colitic, but not normal, uninflamed mice (Figure S2A). CD4^+^CD25^+^ Treg cells are known to inhibit colitis and maintain intestinal homeostasis (Read et al., 2000) (Figure 1A). In mice protected from colitis by Treg cells, there was no accumulation of E-cadherin^+^ DCs above those found in the GALT of unreconstituted *Rag2*^−/−^ mice (Figures 1C and 1D). Quantitative PCR (qPCR) analysis confirmed DC E-cadherin expression at the mRNA level (Figure S2B).

To assess the role of T cell-derived signals in the accumulation of E-cadherin^+^ DCs, we utilized an anti-CD40 model of innate colitis. In this model, injection of an agonistic CD40 mAb leads to focal colitis with a large accumulation of activated DCs in the MLN and colon. We found that approximately 85% of MLN and 50% of colonic DCs expressed E-cadherin (Figure 1E). By contrast, despite a large systemic and intestinal inflammatory response, *Helicobacter hepaticus*-induced innate colitis was not associated with an increase in the frequency of E-cadherin^+^ DCs in either the GALT or spleen (Figure 1F). Together, these data indicate that the accumulation of E-cadherin^+^ DCs is linked to T cells and that the provision of a CD40 signal alone is sufficient to drive accumulation of E-cadherin^+^ DCs.

### Phenotypic Characterization of E-Cadherin^+^ DCs during Intestinal Inflammation

**Figure 2:**
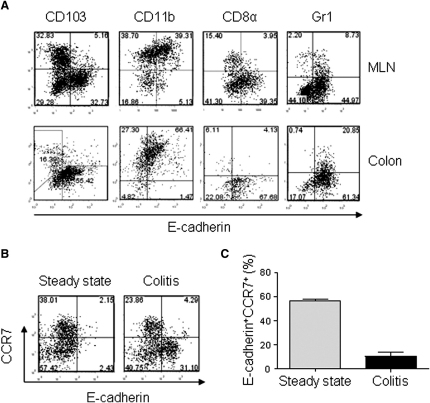
Phenotypic Characterization of E-Cadherin^+^ DCs during Inflammation (A) Staining of CD103, CD11b, CD8α, Gr1, and E-cadherin gated on CD11c^hi^ DCs from MLN and colon cells prepared from colitic BALB/c *Rag2*^−/−^ mice. Positioning of the quadrants reflects the isotype control. Experiment was repeated more than two times. In all cases, representative plots from three to five individual analyses are shown. (B and C) MLN cells were prepared from BALB/c WT and colitic BALB/c *Rag2*^−/−^ mice. (B) Representative staining of CCR7 and E-cadherin gated on CD11c^hi^ DCs. (C) Mean percentage of E-cadherin^+^CD11c^hi^ cells expressing CCR7. Data are representative of two independent experiments. Error bars represent SD. (n = 4–6 mice per group). See also Figure S2.

Phenotypic characterization showed that during colitis, the majority of MLN and colon E-cadherin^+^ DCs were CD103^−^CD11b^+^CD8α^−^Gr1^−^ (Figure 2A). Under inflammatory conditions, E-cadherin^+^ DCs did not express CCR7 (Figures 2B and 2C), suggesting that these cells may enter the MLN directly from the blood (Jang et al., 2006).

### E-Cadherin^+^ DCs Arise from Inflammatory Gr1^+^ Monocytes

**Figure 3:**
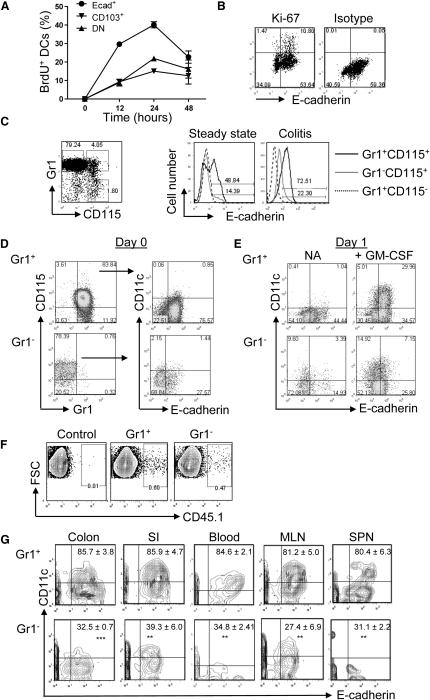
Gr1^+^ Monocytes Differentiate into E-Cadherin^+^ DCs (A) Colitic BALB/c *Rag2*^−/−^ mice were injected i.p. with 2 mg of BrdU. At various time points, whole colon cells were stained for E-cadherin, CD103 and CD11c surface markers, and for intracellular BrdU. Cells were gated on the CD11c^hi^ population and then examined for the percentage of BrdU^+^ cells among each DC subset. Each symbol represents three mice assayed at each time point. (B) Representative staining of E-cadherin, Ki-67, and the relevant isotype control of Ki-67 on gated CD11c^hi^ cells from MLN cell preparations from colitic mice (n = 4). (C) Expression of E-cadherin by CD115^+^Gr1^+^ and Gr1^−^ monocytes taken from BM of WT and colitic *Rag2*^−/−^ mice. Gates applied shown on left panel. Black solid line, E-cadherin expression by Gr1^+^CD115^+^ cells; gray solid line, E-cadherin expression by Gr1^−^CD115^+^ cells; dashed line, E-cadherin expression by Gr1^+^CD115^−^ control cells. Representative plots from three to five individual analyses are shown. (D) Representative staining of E-cadherin and CD11c on flow cytometry-sorted Gr1^+^ and Gr1^−^ CD115^+^ monocytes from pooled BM of colitic BALB/c *Rag2*^−/−^ mice (n = 8). (E) The sorted monocytes were cultured overnight in media with or without GM-CSF, and the next day, the cells were harvested and analyzed for E-cadherin and CD11c expression. (F and G) 3 × 10^6^ sorted Gr1^+^ or Gr1^−^ CD11b^+^ckit^−^CD11c^−^CD115^+^ monocytes from pooled BM and blood of naive B6 CD45.1 mice were injected i.v. into colitic B6 CD45.2 *Rag1*^−/−^ mice, and 36 hr later, various tissues were harvested. Cells were gated on the CD45.1 population, and E-cadherin expression among the CD45.1^+^CD11c^hi^ cells was examined. Cells from nontransferred control mice were isolated in parallel. (F) Representative CD45.1 staining on MLN cells. (G) Staining of E-cadherin and CD11c gated on CD45.1^+^ cells from colitic mice that received Gr1^+^(top panel) or Gr1^−^ (bottom panel) CD115^+^ monocytes (n = 3 per group). Numbers represent mean ± SD of CD11c^hi^ cells expressing E-cadherin. Representative data from one of three experiments; similar experiments gave the same results. See also Figure S3.

To investigate the lineage relationship of E-cadherin^+^ DCs to other intestinal DC subsets, bromodeoxyuridine (BrdU)-labeling studies were performed (Figure 3A). DCs were divided into three subsets based on E-cadherin and CD103 expression: E-cadherin^+^CD103^−^; CD103^+^E-cadherin^−^; and E-cadherin^−^CD103^−^ double negative (DN) cells. Each of these subsets showed rapid BrdU uptake, without any detectable lag in the labeling kinetics, which indicated that the E-cadherin^+^ DCs were not derived from either the CD103^+^E-cadherin^−^ or the DN cell populations. Furthermore, the E-cadherin^+^ DCs showed the fastest input of labeled cells, suggesting recent generation from a dividing precursor. Indeed, we also found that approximately 20% of MLN E-cadherin^+^ DCs expressed Ki-67 (Figure 3B), indicating that this newly differentiated DC population may undergo a limited number of cell divisions.

After emerging from the bone marrow (BM) Gr1^+^CCR2^+^CX_3_CR1^lo^CD115^+^ monocytes home to sites of inflammation, where they differentiate into inflammatory DCs (Geissmann et al., 2003). We next determined whether E-cadherin^+^ DCs were monocyte derived. We found that during both steady-state and inflammatory conditions, the majority of Gr1^+^CD11b^+^CD115^+^ monocytes themselves expressed E-cadherin (Figure 3C). A small population of Gr1^−^CD11b^+^CD115^+^ cells was also E-cadherin^+^. To investigate whether these progenitor cells could give rise to E-cadherin^+^ DCs, CD115^+^ BM monocytes were sorted by flow cytometry into Gr1^+^ and Gr1^−^ fractions and stimulated overnight with GM-CSF. Gr1^+^ monocytes retained their expression of E-cadherin and upregulated CD11c to elicit a population of E-cadherin^+^CD11c^+^ DCs (Figures 3D and 3E). Similar experiments were also performed in the presence of Flt3L but neither of the monocyte subsets differentiated into E-cadherin^+^CD11c^+^ cells (data not shown).

To determine whether Gr1^+^ monocytes also differentiated into E-cadherin^+^ DCs in vivo, adoptive transfer experiments were performed. Flow cytometry-sorted Gr1^+^ and Gr1^−^ CD115^+^CD11b^+^ckit^−^CD11c^−^ BM monocytes from CD45.1 mice (Figure S3A) were injected i.v. into colitic CD45.2 recipients. After 36 hr, CD45.1 cells present in various tissues (Figure 3F) were examined for their expression of E-cadherin and CD11c. Gr1^+^ monocytes differentiated into CD11c^hi^ cells in all tissues examined, and the vast majority of these expressed E-cadherin (Figure 3G). There was no evidence that E-cadherin^+^CD11c^+^ cells in the MLN derived from the colon because there was a moderate increase in the proportion of CD45.1^+^Gr1^+^ monocyte-derived CD11c^hi^ cells in the MLN compared to the colon (Figure S3B). By contrast, Gr1^−^ monocytes were significantly reduced in their ability to generate E-cadherin^+^CD11c^hi^ cells (Figure 3G). These data suggest that during colitis, Gr1^+^, and to some degree Gr1^−^, monocytes may independently home to sites of inflammation and differentiate into E-cadherin-expressing CD11c^hi^ cells.

### TGF-β Limits the Accumulation of E-Cadherin^+^ DCs

**Figure 4:**
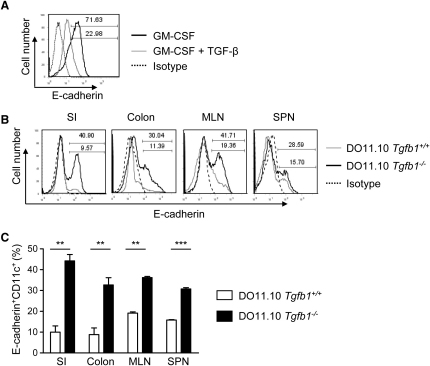
TGF-β Limits the Accumulation of E-Cadherin^+^ DCs (A) Day 8 BM-DCs cultured with GM-CSF in the absence (solid black line) or presence (solid gray line) of TGF-β were stained for E-cadherin and CD11c. Histogram shows representative staining of E-cadherin on CD11c^hi^ gated cells. Dashed line represents the isotype control. (B) Representative staining of E-cadherin on CD11c^hi^ gated cells taken from DO11.10 *Tgfb1*^−/−^ mice (black line) and littermate controls (gray line). Dashed line represents the isotype control. (C) Mean percentages of CD11c^hi^ cells expressing E-cadherin from the small intestine, colon, MLN, and spleen of DO11.10 *Tgfb1*^−/−^ mice (filled bars, n = 3) and littermate controls (open bars, n = 4). Error bars represent SD. Representative data from one of three independent experiments.

It is well established that TGF-β plays an important role in intestinal homeostasis and has direct regulatory effects on DCs (Zhang et al., 2004). In light of these findings, we examined the effect of TGF-β treatment on in vitro-generated BM-DCs. We found that the addition of TGF-β led to a profound downregulation of E-cadherin expression by the BM-DCs (Figure 4A). Furthermore, in TGF-β-deficient mice, which do not display histological signs of colitis, we found enhanced frequencies of E-cadherin^+^ DCs compared to littermate controls (Figures 4B and 4C). This increase was most pronounced in the colon and small intestine (SI), indicating that tissue-specific regulatory cytokines, such as TGF-β, may be important for limiting the accumulation of E-cadherin^+^ DCs.

### E-Cadherin^+^ DCs Display a Proinflammatory Gene Expression Profile

**Figure 5:**
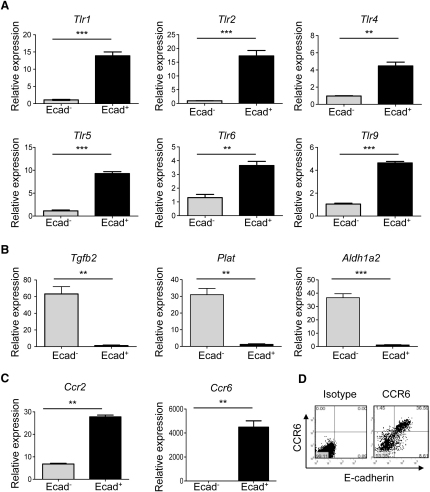
E-Cadherin^+^ DCs Display a Proinflammatory Gene Expression Profile E-cadherin^+^ and E-cadherin^−^ CD11c^hi^CD4^−^7AAD^−^CD103**^−^** cells were flow cytometry-sorted from pooled MLN cell preparations taken from colitic BALB/c *Rag2*^−/−^ mice (n = 10). (A) *Tlr*, (B) *Tgfb2*, *Plat*, and *Aldh1a2*, and (C) *Ccr2* and *Ccr6* gene expression was assayed in triplicate by qPCR, and each sample was normalized relative to HPRT expression. Error bars represent SEM of triplicate samples. (D) *Ccr6* gene expression was confirmed by flow cytometry analysis on MLN cells taken from colitic BALB/c *Rag2*^−/−^ mice. Data show representative E-cadherin and CCR6 staining on CD11c^hi^ gated cells. Data are representative from one of three independent experiments. See also Figure S4.

To gain insight into the function of E-cadherin^+^ DCs, we performed gene expression profiling by qPCR. We have previously shown that CD103^−^ DCs produce inflammatory cytokines upon activation (Coombes et al., 2007); however, in these studies, E-cadherin expression among the DCs was not considered. Subsequently, for the following experiments, E-cadherin^−^CD103^−^ DCs were chosen as the reference population with which comparisons were made. We found that E-cadherin^+^ CD11c^hi^CD103^−^7-AAD^−^CD4^−^ DCs expressed more than 5-fold higher amounts of various toll-like receptors mRNA than E-cadherin^−^ counterparts (Figure 5A), but E-cadherin^+^ DCs did not express *Tgfb2*, *Plat*, or *Aldh1a2*, which are genes associated with a tolerogenic DC signature (Figure 5B). It is now well established that CCR2 is required for the recruitment of monocytes into inflamed tissues (Dunay et al., 2008) and that CCR6 expression marks a subset of inflammatory DCs within the SI (Salazar-Gonzalez et al., 2006). We found that E-cadherin^+^ DCs expressed approximately 30-fold higher amounts of *Ccr2* and 3,000-fold higher amounts of *Ccr6* mRNA than E-cadherin^−^ DCs, and flow cytometry analysis confirmed that E-cadherin^+^ DCs were indeed the main expressers of CCR6 in the MLN (Figures 5C and 5D). These data suggest that E-cadherin^+^ DCs may be hyper-responsive to microbial stimulation, reinforcing their inflammatory potential.

### Functional Characterization of E-Cadherin^+^ DCs

To confirm that MLN E-cadherin^+^CD11c^hi^ cells from colitic mice displayed characteristics associated with DCs, we investigated their phenotypic and functional properties. E-cadherin^+^CD11c^hi^ cells expressed high amounts of MHC class II, CD80, and CD40, but moderate amounts of CD86, compared with E-cadherin^−^ counterparts (Figure S4A). A cardinal feature of DCs is their capacity to process antigen and prime naive T lymphocytes (Banchereau et al., 2000). We thus compared the ability of MLN E-cadherin^+^ and E-cadherin^−^ CD11c^hi^CD103^−^7-AAD^−^CD4^−^ cells that were pulsed with OVA protein to activate splenic CFSE-labeled CD4^+^ T cells isolated from DO11.10 SCID mice. Antigen stimulation by both DC populations led to increased T cell activation, as assessed by cell division, compared with myeloid MHC class II^+^CD11c^−^F4/80^+^ control cells (Figure S4B). Importantly, E-cadherin^+^ DCs also supported higher CD4^+^ T cell accumulation than E-cadherin^−^ DCs or myeloid control cells (Figure S4C). Together, these data show that E-cadherin^+^CD11c^hi^ cells from the MLN are capable of antigen uptake, processing, and presentation, leading to the antigen-induced activation and accumulation of naive T cells. Based on these criteria, E-cadherin^+^CD11c^hi^ MLN cells bear the hallmark features of DCs.

### E-Cadherin^+^ DCs Produce Colitogenic Cytokines upon Activation

**Figure 6:**
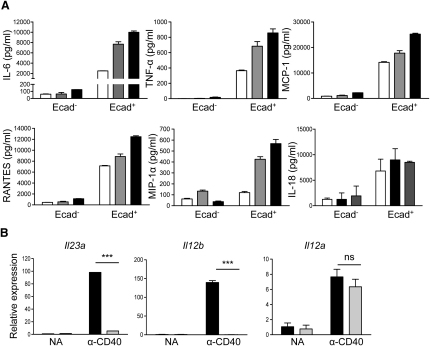
E-Cadherin^+^ DCs Produce Proinflammatory Cytokines and Chemokines E-cadherin^+^ and E-cadherin^−^ CD11c^hi^CD103^−^7-AAD^−^CD4^−^ DCs were sorted from pooled MLN cell preparations taken from colitic BALB/c *Rag2*^−/−^ mice (n = 10). The DCs were cultured in triplicate overnight in media alone or in the presence of 10 μg/ml anti-CD40 or 1 μg/ml LPS. (A) Supernatants were harvested, and cytokine and chemokine concentrations were analyzed by FlowCytomix immunoassays (open bars, no additions; gray bars, anti-CD40; black bars, LPS). (B) *Il23a* (*Il-23p19*), *Il12b* (*Il-12* and *Il-23 p40*), and *Il12a* (*Il-12p35*) gene expression was assayed by qPCR, and each sample was normalized relative to HPRT expression (black bars, E-cadherin^+^ DCs; gray bars, E-cadherin^−^ DCs). Error bars represent SEM of triplicate samples. Data are representative from one of three independent experiments.

To further investigate the function of this DC population, E-cadherin^+^ and E-cadherin^−^ CD11c^hi^CD103^−^7-AAD^−^CD4^−^ DCs from the MLN of colitic mice were stimulated overnight with LPS or an agonistic CD40 mAb, and cytokine production was examined. Even in the absence of further stimulation, E-cadherin^+^, but not E-cadherin, DCs produced high amounts of proinflammatory cytokines and chemokines, which could be enhanced by the addition of either anti-CD40 or LPS (Figure 6A). By qPCR, we also found that upon stimulation through CD40, E-cadherin^+^ DCs expressed more than 100-fold higher amounts of IL-23p19 and IL-12p40 mRNA than E-cadherin^−^ DCs, with only a comparatively moderate increase in the expression of IL-12p35 (Figure 6B). These data suggest that E-cadherin^+^ DCs may function as effector leukocytes in vivo, involved in proinflammatory cytokines and chemokines upon activation.

### E-Cadherin^+^ DCs Exacerbate T Cell-Mediated Colitis

**Figure 7:**
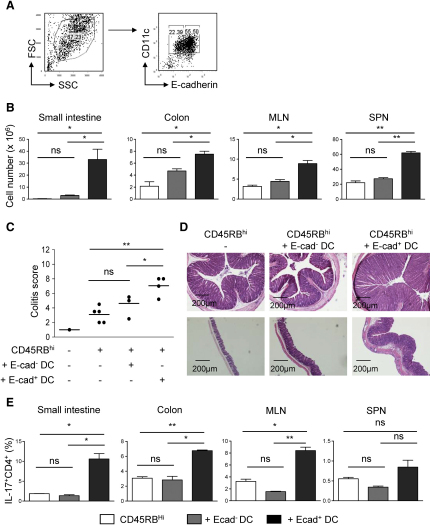
Adoptive Transfer of E-Cadherin^+^ BM-DCs Exacerbates T Cell-Mediated Colitis (A–E) BM-DCs were generated in the presence of GM-CSF. At day 8 of culture, cells were harvested, E-cadherin^+^ and E-cadherin^−^ BM-DCs were sorted, and 3 × 10^6^ cells were i.p. injected into B6 *Rag1^−/−^* mice that had received CD4^+^CD45RB^hi^ T cells 14 days earlier. Ten days post-DC transfer, the mice were sacrificed and examined for exacerbated colitis compared to non-DC transferred controls. (A) Gating strategy applied to sorting E-cadherin^+^ and E-cadherin^−^ BM-DCs. (B) Total cell numbers of small intestine, colon, MLN, and spleen preparations. (C) Colon histology scores. (D) Representative photomicrographs of hematoxylin and eosin-stained sections of colon (top panel) and caecum (bottom panel). (E) Mean percentages of CD4^+^ T cells expressing IL-17 in the small intestine, colon, MLN, and spleen. Open bars, CD45RB^hi^ T cells; gray bars, CD45RB^hi^ T cells and E-cadherin^−^ BM-DCs; black bars, CD45RB^hi^ T cells and E-cadherin^+^ BM-DCs. Error bars represent SD. Data shown are representative of one out of three independent experiments. See also Figure S5.

To assess the functional relevance of E-cadherin^+^ DCs in vivo, we examined the effect of their administration on the progression of T cell-mediated colitis. As GM-CSF promoted the generation of E-cadherin^+^ BM-DCs (Figure S5A), which are generally considered to represent an immature DC population (Inaba et al., 1992a), we postulated that these cells may be more effective than ex vivo-derived DCs at migrating to the intestine. This system also allows the generation of high DC yields, which are otherwise a limiting factor. Furthermore, E-cadherin^+^ BM-DCs and MLN DCs share many similar phenotypic characteristics, including high MHC class II and costimulatory molecule expression, and the majority of both E-cadherin^+^ BM-DCs and MLN DCs are CD103^−^, CD8α^−^, and CD11b^+^ (Figures S5B–S5D). Consequently, flow cytometry-sorted BM-derived E-cadherin^+^ and E-cadherin^−^ CD11c^hi^ cells (Figure 7A) were injected i.p into B6 *Rag1*^−/−^ mice that had received naive CD4^+^CD45RB^hi^ T cells 2 weeks earlier. The intestinal inflammatory response was examined 10 days later. Strikingly, mice transferred with E-cadherin^+^, but not E-cadherin^−^, BM-DCs displayed a significant increase in total cell numbers in the colon and GALT (Figure 7B) and developed more severe intestinal pathology than control groups (Figure 7C). This was associated with severe inflammation of the caecum and the development of crypt abscesses and submucosal colonic inflammation, the latter of which are parameters of severe colitis (Figure 7D).

We investigated the effect of BM-DC transfer on the T lymphocyte compartment of the GALT. Both E-cadherin^+^ and E-cadherin^−^ BM-DCs failed to promote enhanced frequencies of IFN-γ^+^CD4^+^ T cells and no differences were observed in the frequency of Treg cells (data not shown). However, recipients of E-cadherin^+^, but not E-cadherin^−^, BM-DCs displayed significantly higher frequencies of IL-17^+^CD4^+^ T cells both in the intestine and GALT (Figure 7E). Furthermore, due to the overall increase in total cell numbers, E-cadherin^+^, but not E-cadherin^−^, BM-DCs also generated enhanced numbers of IL-17^+^ and IFN-γ^+^CD4^+^ T cells (data not shown). Taken together, these results strongly suggest that E-cadherin marks a subset of physiologically relevant inflammatory DCs that are able to exacerbate T cell-mediated colitis.

## Discussion

Here, we showed that the ligand for CD103, E-cadherin, marks a population of CD11c^hi^ cells that accumulates in the GALT and colon of colitic mice. E-cadherin^+^ DCs can derive from Gr1^+^ inflammatory monocytes, they express an array of TLRs, and upon activation, they produce high amounts of proinflammatory effector cytokines. Furthermore, transfer of E-cadherin^+^ DCs to immunodeficient mice exacerbates T cell-driven mucosal immune pathology. These results newly identify E-cadherin as a marker of monocyte-derived inflammatory DCs that may contribute to the pathogenesis of chronic T cell-mediated colitis.

Tissues contain distinct DC subsets that vary from one another according to ontogeny, receptor expression, and temporal separation in order to carry out distinct functions (Merad and Manz, 2009). This division of labor between tissue resident and inflammatory DCs may provide a mechanism by which tolerogenic and effector responses can be accommodated. DCs with an inflammatory phenotype accumulate in response to infection. Thus, TNF and iNOS-producing (Tip) DCs were shown to produce nitric oxide and TNF-α in response to *Listeria* and *Toxoplasma* infection (Serbina et al., 2003). In addition, CCR6^+^ DCs are rapidly recruited within the follicle-associated epithelium of the SI Peyer's Patches after oral *Salmonella* infection (Salazar-Gonzalez et al., 2006). Monocytes are the major precursors of inflammatory DCs (Geissmann et al., 2003). Gr1^+^CCR2^+^CX_3_CR1^lo^ monocytes emigrate from the BM in response to infection and home to sites of inflammation, where they are able to differentiate into DCs (Serbina and Pamer, 2006). Blood-derived Gr1^+^ inflammatory DCs also mediate acute Th1 responses to viral infections in vivo (Nakano et al., 2009). Here, we showed that E-cadherin^+^ DCs share several phenotypic and functional properties with these previously described inflammatory DC populations.

The two main DC subsets present in the intestinal lamina propria derive from distinct cell lineages, which may account for their diverse functional properties. CD103^+^CX3CR1^−^ DCs develop from pre-DCs, whereas Gr1^+^ monocytes, under the control of *m*acrophage-*c*olony *s*timulating *f*actor (M-CSF) and to a lesser extent Flt3L, give rise to CD103^−^CD11b^+^CX_3_CR1^+^ LP DCs (Bogunovic et al., 2009; Varol et al., 2009). We and others have shown that CD103^+^ DCs promote the generation of Treg cells (Coombes et al., 2007; Sun et al., 2007); however, much less is known about the function of the reciprocal CD103^−^ DC population (Fortin et al., 2009). Varol et al. (2009) showed that monocyte-derived CD103^−^CX_3_CR1^+^ DCs are sufficient to reconstitute acute colitis in DC-depleted mice. Several lines of evidence suggest that Gr1^+^ monocytes are also the predominant precursor of inflammatory E-cadherin^+^ DCs. First, BrdU labeling studies showed that E-cadherin^+^ DCs are not derived from either CD103^+^ or CD103^−^E-cadherin^−^ DC subsets. A large proportion of E-cadherin^+^ DCs incorporated BrdU over 12 hr, and approximately 20% of these cells proliferated in situ, suggesting their recent derivation from a proliferating precursor. This is likely to be an inflammatory blood monocyte as Gr1^+^, and to a lesser extent Gr1^−^, monocytes expressed E-cadherin. Second, adoptively transferred Gr1^+^ monocytes migrated to sites of inflammation and gave rise to E-cadherin^+^ DCs within 48 hr. Gr1^+^ monocytes preferentially accumulated in the MLN over the colon, which suggests that circulating monocytes may independently seed both of these sites, giving rise to E-cadherin^+^CD11c^hi^ cells. In support of this, under inflammatory conditions, the majority of MLN E-cadherin^+^ DCs did not express CCR7, suggesting they are not a migratory population. By contrast, they did express high CCR2 and CCR6. CCR2 has previously been implicated in the egression of monocytes from the BM (Serbina and Pamer, 2006), whereas CCR6 is required for the localization of monocyte-derived DCs within the SI PPs (Cook et al., 2000).

In the noninflamed intestine, E-cadherin^+^ DCs represent only a minor population of the total DC compartment. In colitic mice, they dramatically increase in frequency, making up to 50% of CD11c^hi^ cells in the colon and MLN. These cells are likely to contribute to disease pathogenesis as adoptive transfer of in vitro generated E-cadherin^+^ DCs into T cell-restored immunodeficient hosts led to an increased Th17 cell response and enhanced colitis. E-cadherin^+^ DCs express a number of features that may contribute to their pathogenic action in the gut. They express high amounts of a number of TLRs, making them poised to respond to microbial triggers. Upon activation, E-cadherin^+^ DCs produce large quantities of proinflammatory cytokines, such as IL-6, TNF-α, and IL-23 p19, that have previously been implicated in the pathogenesis of IBD (Hue et al., 2006; Uhlig et al., 2006b). Even in the absence of further stimulation, E-cadherin^+^ DCs produce considerable amounts of proinflammatory chemokines, such as MCP-1 and RANTES. In the intestine or MLN, E-cadherin^+^ DCs may therefore function as cytokine-chemokine reservoirs, which upon activation, release their cargo and, in doing so, rapidly create chemotactic gradients that could serve to recruit effector monocytes, neutrophils, and T cells to the site of inflammation and, hence, propagate the immune cascade. Furthermore, E-cadherin^+^ DCs are capable of activating naive T cells and may directly promote Th17 cells in the MLN through their production of IL-23 and IL-6. As E-cadherin^+^ DCs represent a recently recruited monocyte-derived DC population, they may escape gut conditioning, endowing them with the capacity to override the suppressive gut environment and, hence, stimulate inflammatory responses.

Although E-cadherin^+^ monocytes are present in the steady-state BM, the accumulation of E-cadherin^+^ DCs in the colon and GALT is a feature of T cell-mediated, but not bacteria-induced, innate colitis. Colitogenic T cells express high levels of CD40L and disruption of CD40-CD40L interactions inhibits T cell transfer colitis (De Jong et al., 2000). E-cadherin^+^ DCs express high amounts of CD40, and addition of an agonistic CD40 mAb is sufficient to induce colitis in B6 *Rag1*^−/−^ mice, which is associated with the marked accumulation of E-cadherin^+^ DCs in the intestine and MLN. These results suggest that CD40 signaling in the absence of further T cell-derived signals is sufficient to lead to the recruitment and differentiation of inflammatory monocytes into E-cadherin^+^ DCs, and it raises the possibility that E-cadherin^+^ DCs play a key role in driving anti-CD40-mediated colitis. In the T cell transfer model of colitis, CD40L^+^ T cells may act in a positive feedback loop to amplify the accumulation and activation of E-cadherin^+^ inflammatory DCs. In addition, activated T cells may provide growth factors, such as GM-CSF or M-CSF, which could drive the development of E-cadherin^+^ DCs.

Signaling through E-cadherin expressed on in vitro-generated LCs inhibits DC maturation and, subsequently, proinflammatory cytokine production (Riedl et al., 2000). More recently, it has been shown that disruption of E-cadherin-mediated DC clustering generates tolerogenic DCs, a process dependent on the downstream signaling molecule β-catenin (Jiang et al., 2007). The expression of E-cadherin on inflammatory monocytes and monocyte-derived DCs may therefore provide a negative feedback loop to control inflammation-induced DC maturation in tissues. Intestinal Treg cells and tolerogenic DCs in the intestine express CD103, making it tempting to speculate that CD103-E-cadherin interactions may contribute to Treg cell mediated immune suppression. Indeed, because E-cadherin^+^ DCs fail to accumulate in the steady state and during Treg cell-mediated suppression of colitis, Treg cells may control the accumulation of inflammatory E-cadherin^+^ DCs from monocyte precursors. Notably, Treg cells were unable to prevent T cell mediated colitis in CD103-deficient recipients, suggesting a functional role for CD103^+^ DCs in maintaining intestinal immune homeostasis (Annacker et al., 2005).

Little is known about the factors that control E-cadherin expression on inflammatory DCs. IL-1β and TNF-α downregulate E-cadherin expression on human monocyte-derived LCs, however the in vivo relevance of this was not established (Jakob and Udey, 1998). As observed in neoplastic cells (Fransvea et al., 2008), we found that TGF-β downregulates E-cadherin expression on BM-DCs cultured with GM-CSF. TGF-β also controls the accumulation of E-cadherin^+^ DCs in vivo as DO11.10 *Tgfb1*^−/−^ mice exhibited increased frequencies of E-cadherin^+^ DCs in the intestine. Similarly, IL-10 prevents intestinal inflammation through effects on myeloid cells (Takeda et al., 1999) and has also been shown to limit TipDC-mediated pathogenicity (Guilliams et al., 2009). Both TGF-β and IL-10 are important components of the immune suppressive network in the gut, and control of the generation or recruitment of inflammatory DCs may contribute to their function.

In summary, we have identified E-cadherin as a marker of a subset of inflammatory monocyte-derived DCs that contribute to chronic T cell-mediated intestinal inflammation. Further understanding of the factors that control the migration and function of these cells may provide novel therapeutic strategies in IBD.

## Experimental Procedures

### Mice

BALB/c, BALB/c *Rag2*^−/−^, DO11.10 *Tgfb1*^−/−^, C57BL/6 (B6) WT, B6 CD45.1, B6 *Rag1*^−/−^, 129SvEv *Rag2*^−/−^ and DO11.10 SCID TCR-transgenic mice were bred and maintained under specific pathogen-free conditions in an accredited animal facility at the University of Oxford. All procedures involving animals were conducted according to the requirements and with the approval of the United Kingdom Home Office Animals (Scientific Procedures) Acts, 1986.

### Antibodies

The following antibodies were used for CD4^+^ T cell purification: anti-mouse CD8 (YTS169), -MHC class II (TIB120), -Mac-1 (M1/70), and -B220 (RA3-6B2, all purified from hybridoma supernatant by affinity chromatography). The following anti-mouse monoclonal antibodies were used for multiparameter FACS analyses: CD45RB (16A), CD25 (PC61), CD80 (16-10A1), CD86 (GL1), CD40 (3/23), CD11b (M1/70), CD8α (53-6.7), CCR7 (4B12), CCR6 (140706), CD11c (HL3), CD103 (M290), CD4 (GK1.5/RM4-5), 7-AAD, CD3e (145-2C11), CD115 (AF598), E-cadherin (36), Gr-1 (RB6-8C5), I-A^b^ (2G9), and Ki-67 (B56). The corresponding isotype controls and the secondary reagent allophycocyanin-Cy7-conjugated streptavidin were all purchased from BD Biosciences. For use in both in vivo and in vitro cultures, anti-mouse CD40 (FGK45) were purified from hybridoma supernatant by affinity chromatography and shown to contain <0.1 EU endotoxin per milligram of protein.

### Preparation and Culture of CD11c^hi^ Subsets

Spleen and lymph nodes were digested with collagenase type VIII (Sigma-Aldrich) as previously described (Coombes et al., 2007). CD11c^+^ cells were then positively selected on an LS MACS column (Miltenyi Biotec). MLN cell suspensions from colitic mice were not CD11c-enriched because of the local accumulation of DCs during inflammation. Cell suspensions were prepared from the colon and SI LP as previously described (Sun et al., 2007; Uhlig et al., 2006a). For FACS analysis, we first gated on the high forward and side scatter cells in order to remove the majority of CD11c^−^ cells, 7-AAD^+^ dead cells were excluded, and E-cadherin expression among the CD11c^hi^ cells was examined. For cell sorting, cells were purified into E-cadherin^+^ and E-cadherin^−^ CD11c^hi^CD103^−^CD4^−^7-AAD^−^ fractions (>98%) on a MoFlo sorter (Dako cytomation). For analysis of cytokine production, DC subsets were cultured overnight in DMEM supplemented with 10% FCS, 2 mM L-glutamine, and 100 U of penicillin and streptomycin. LPS (1 μg/ml, Sigma-Aldrich) or anti-CD40 (10 μg/ml) were added to some wells. Supernatants were analyzed for chemokine and cytokine production using a FlowCytomix immunoassay (Bender MedSystems), according to the manufacturer's instructions. Cells were prepared for qPCR analysis, as described below.

### T Cell Transfer Model of Colitis

Naive CD4^+^CD25^−^CD45RB^hi^ T cells were isolated from B6 or BALB/c WT mice and were FACS sorted as previously described (Read et al., 2000). Sex-matched B6 *Rag1^−/−^* or BALB/c *Rag2*^−/−^ recipients were i.p. injected with 4 × 10^5^ cells, and the development of intestinal inflammation was monitored. Mice were killed >6 weeks after cell transfer, and tissues were collected for cell populations or histological analysis. For some experiments, CD4^+^CD25^+^CD45RB^lo^ T cells were sorted and cotransferred with the naive T population at 2 × 10^5^ cells per mouse.

### Anti-CD40 Model of Colitis

B6 *Rag1*^−/−^ mice were i.p. injected with 200 μg FGK45 CD40 mAb. Mice were weighed daily and killed at day 7.

### *Helicobacter hepaticus* Induced Model of Colitis

129SvEv *Rag2*^−/−^ mice were inoculated intragastrically with 0.3 ml of a *Helicobacter hepaticus* suspension (NCI-Frederick isolate 1A) prepared to a McFarland turbidity standard of 1.0 in PBS representing 2.45 × 10^9^ CFU/ml. Three doses were administered on days 0, 2, and 4, and mice were killed 6 weeks later.

### Assessment of Pathology

Mice were killed when clinical symptoms of intestinal inflammation (diarrhea and weight loss) became apparent, usually >6 weeks after initiation of an experiment. Samples of distal, mid-, and proximal colon were immediately fixed in buffered 10% formalin. Paraffin embedded 5 μm sections were stained with hematoxylin and eosin, and inflammation was assessed using a previously described scoring system (Izcue et al., 2008).

### BrdU Labeling and Analysis

BALB/c *Rag2*^−/−^ mice were transferred with CD4^+^CD45RB^hi^ T cells and after 6 weeks, when the recipients displayed clinical signs of intestinal inflammation, the mice were i.p. injected with 2 mg BrdU (Sigma-Aldrich) resuspended in PBS. Cell suspensions were prepared at different time points after BrdU administration and were analyzed for BrdU incorporation with the BrdU Flow Kit (BD Biosciences) according to the manufacturer's instructions.

### Culture and Transfer of Monocytes

BM and whole blood cell suspensions were isolated from B6 and BALB/c WT or colitic B6 *Rag1^−/−^* and BALB/c *Rag2*^−/−^ mice. Erythrocytes were depleted with ACK lysis buffer, and contaminating platelets in the blood were removed by an additional ficoll gradient. For adoptive transfer experiments, FACS-sorted CD115^+^ckit^−^CD11c^−^CD11b^+^ Gr1^+^ or Gr1^−^ monocytes from B6 CD45.1 mice were i.v. injected at 3 × 10^6^ cells into colitic B6 CD45.2 *Rag1^−/−^* mice that had received CD4^+^CD45RB^hi^ T cells >6 weeks earlier. Thirty-six hours after monocyte transfer, tissues were collected for the detection of CD45.1^+^E-cadherin^+^ DCs by FACS analysis. For in vitro culture, FACS-sorted Gr1^+^ or Gr1^−^CD115^+^ monocytes were incubated overnight in RPMI supplemented with 10% FCS, 2 mM L-glutamine, and 100 U of penicillin and streptomycin. GM-CSF (5% hybridoma supernatant) was added to some wells. The cells were analyzed for expression of E-cadherin and CD11c.

### Generation of BM-DCs

BM-DCs were prepared as previously described (Inaba et al., 1992b). Cells were cultured in 6-well tissue culture plates (Costar) for 8 days in complete medium supplemented with 5% GM-CSF. TGF-β (10 ng/ml, R&D systems) was added to some wells from day 0. DCs were collected, incubated with an anti-FcR antibody, and labeled with anti-mouse E-cadherin, CD11c and 7-AAD prior to FACS analysis or cell sorting.

### Quantitation of Gene Expression Using Real-Time PCR

Total RNA was purified from sorted cells using RNAeasy kits (QIAGEN). cDNA was synthesized using Superscript III reverse transcriptase and Oligo dT primers (both from Invitrogen). Quantitative PCR reactions were performed using either quantitect Primer Assays with SYBR green PCR mastermix (QIAGEN) or the following reagents: IL-23 p19 primer AGCGGGACATATGAATCTACTAAGAGA, GTCCTAGTAGGGAGGTGTGAAGTTG, and FAM/TAMRA-labeled probe CCAGTTCTGCTTGCAAAGGATCCGC; IL-12 p40 primers GACCATCACTGTCAAAGAGTTTCTAGAT, AGGAAAGTCTTGTTTTTGAAATTTTTTAA, and FAM/TAMRA-labeled probe CCACTCACATCTGCTGCTCCACAAGAAG; HPRT primers GACCGGTCCCGTCATGC, TCATAACCTGGTTCATCATCGC; and VIC/TAMRA-labeled probe ACCCGCAGTCCCAGCGTCGTC. cDNA samples were assayed in triplicate using a Chromo4 detection system (MJ Research), and gene-expression levels for each individual sample were normalized to HPRT. Mean relative gene expression was determined, and differences were calculated using the 2^−ΔC(t)^ method (Pfaffl, 2001).

### T Cell Differentiation Assay

Cell suspensions were prepared from the spleens of DO11.10-SCID mice. CD4^+^ T cells were isolated by labeling with magnetic anti-CD4 beads (Miltenyi Biotec) and separating the cell populations using LS MACS columns (Miltenyi Biotec). The positively selected CD4^+^ T cells were labeled with 10 μM CFDA-SE using a Vybrant CFDA-SE Cell Tracer Kit (Invitrogen). 2 × 10^5^ T cells were cultured together with 3 × 10^4^ MLN E-cadherin^+^ or E-cadherin^−^ CD11c^hi^CD103^−^7-AAD^−^CD4^−^ cells, or MHC class II^+^CD11c^−^F4/80^+^ cells, in the presence or absence of 5μg/ml OVA protein in complete RPMI for 4 days. T cells were counted and stained for CD4, and T cell proliferation was assessed by dilution of the CFSE dye using FACS analysis.

### Adoptive Transfer of BM-DCs

BM-DCs were generated and sorted into E-cadherin^+^ and E-cadherin^−^ CD11c^hi^ cell fractions. 3 × 10^6^ BM-DC subsets were i.p. injected into B6 Rag1^−/−^ mice that had received CD4^+^CD45RB^hi^ T cells 2 weeks earlier. Ten days post-DC transfer, intestinal tissues were collected for histologic analysis, and intracellular cytokine staining was performed on GALT cell preparations.

### Statistics

An unpaired Student's t test was performed in Prism (Graphpad) in all cases. Where appropriate, mean ± SD is represented on graphs. ^∗^p < 0.05; ^∗∗^p < 0.001; ^∗∗∗^p < 0.001.

## Supplemental Information

Document S1. Five Figures
