# Supplementary material for: E-Cadherin Marks a Subset of Inflammatory Dendritic Cells that Promote T Cell-Mediated Colitis
Source: Immunity. 2010 Apr 23;32(4):557–67. doi: 10.1016/j.immuni.2010.03.017 (PMC2938478; doi:10.1016/j.immuni.2010.03.017)
Supplement: Document S1. Five Figures [file mmc1.pdf]

Immunity, Volume *32*

Supplemental Information

E-Cadherin Marks a Subset of Inflammatory Dendritic Cells that Promote T Cell-Mediated Colitis

Karima RR Siddiqui, Sophie Laffont, and Fiona Powrie

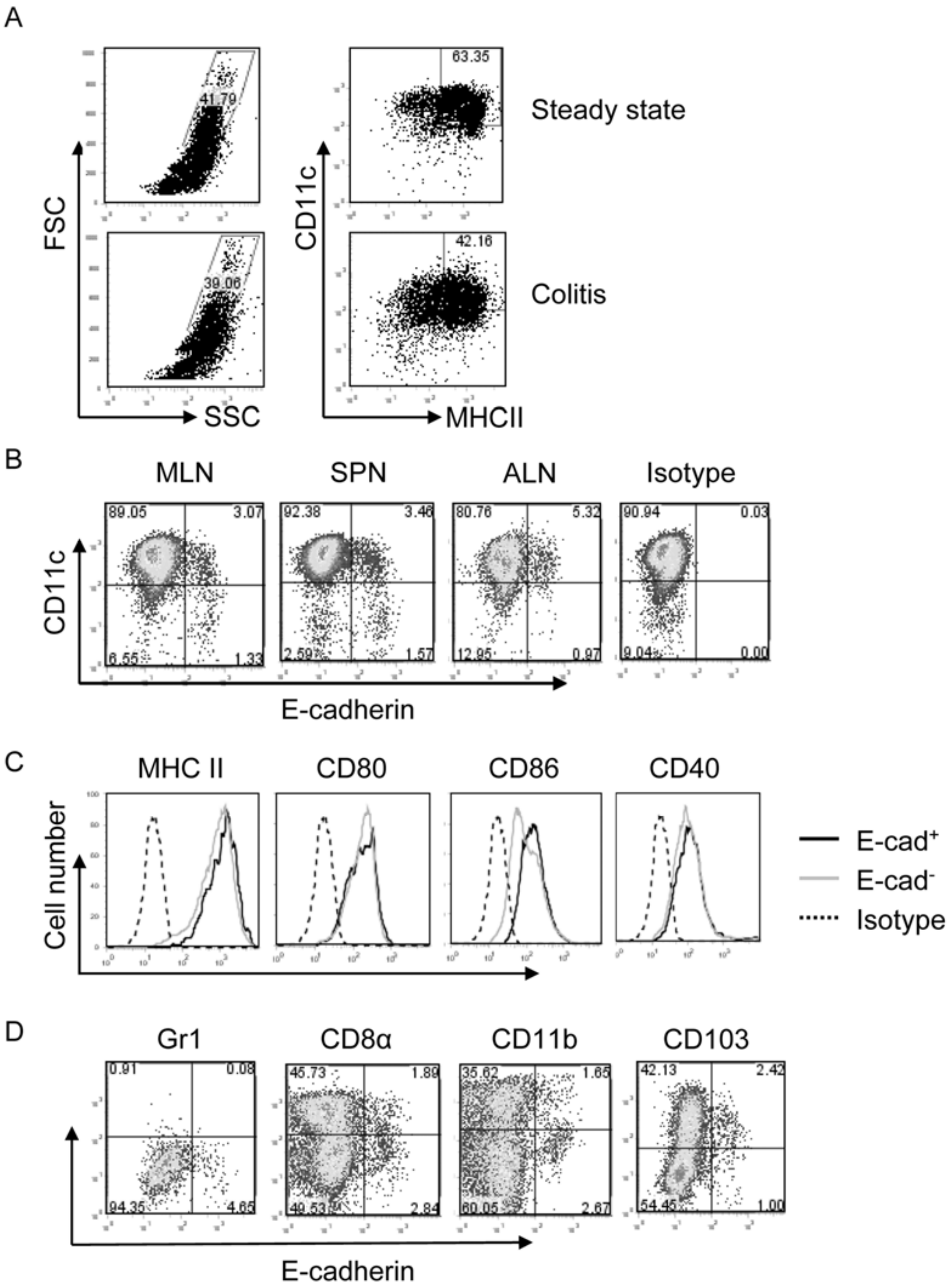
A
41.79
39.06
FSC
SSC
63.35
42.16
CD11c
MHCII
Steady state
Colitis
B
MLN
SPN
ALN
Isotype
89.05
3.07
6.55
1.33
92.38
3.46
2.59
1.57
80.76
5.32
12.95
0.97
90.94
0.03
9.04
0.00
CD11c
E-cadherin
C
MHC II
CD80
CD86
CD40
Cell number
E-cad+
E-cad-
Isotype
D
Gr1
CD8α
CD11b
CD103
0.91
0.08
94.35
4.65
45.73
1.89
49.53
2.84
35.62
1.65
60.05
2.67
42.13
2.42
54.45
1.00
E-cadherin

Figure S1. Related to figure 1. Phenotypic characterisation of E-cadherin$^+$ DCs. Tissues from BALB/c WT mice were harvested and the distribution and phenotype of E-cadherin$^+$ DCs was assessed by FACS. (a) Representative FACS-analysis gating strategies applied to all experiments unless otherwise stated. Steady state, but not colitic, MLN cells were CD11c-enriched. Cells were gated on the high FSC and SSC cell population in order to remove the majority of CD11c$^-$ cells, 7-AAD$^+$ dead cells were excluded and E-cadherin expression amongst the CD11c^{hi}MHC class II^{hi} cells was examined. (b) Staining of E-cadherin, CD11c and the relevant isotype control of E-cadherin on cells from MLN, spleen and ALN (axillary lymph nodes). (c) Expression of MHC class II and co-stimulatory molecules CD80, CD86 and CD40 on E-cadherin$^+$ and E-cadherin$^-$ CD11c^{hi} cells. Black solid lines, E-cadherin$^+$ DCs; grey solid line, E-cadherin$^-$ DCs; and dashed lines, isotype control antibodies. (d) Expression of E-cadherin, Gr1, CD8α, CD11b and CD103 gated on CD11c^{hi} DCs from MLN cell preparations. Positioning of the quadrants reflects isotype controls. In all cases, representative plots from 5-10 individual analyses are shown. Experiments have been repeated more than 3 times.

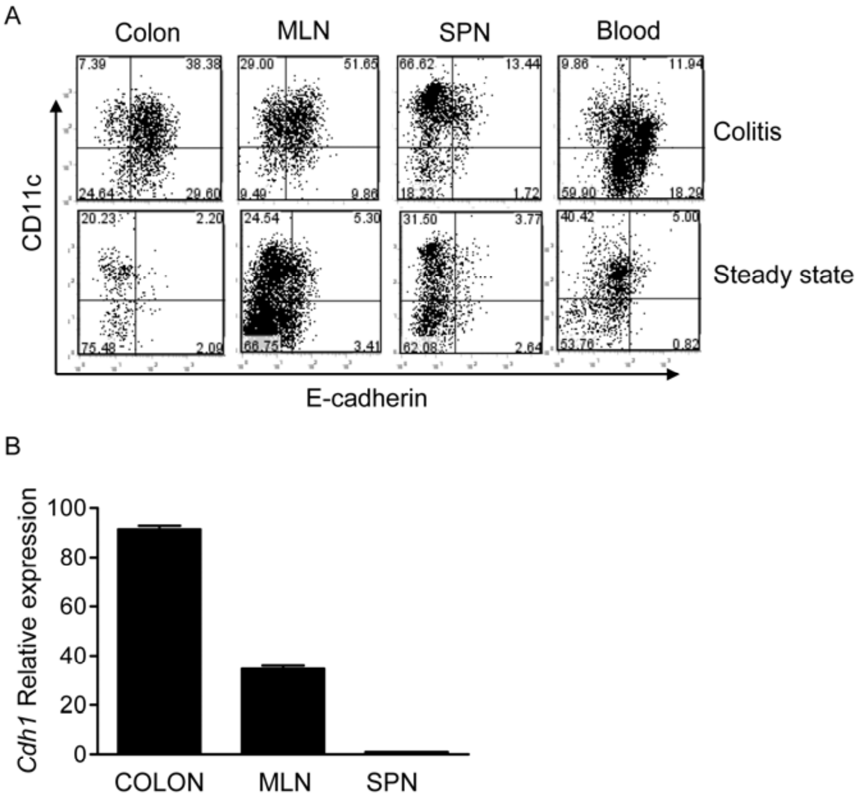

Figure S2. Related to figure 2. E-cadherin expression during T cell mediated colitis

(a) Tissues from colitic B6 $Rag1^{-/-}$ and steady state B6 WT mice were harvested and the distribution of $E\text{-cadherin}^{+}$ DCs was assessed by FACS. Staining of E-cadherin and CD11c gated on MHC class II^{+} cells from colon, MLN, spleen and blood. Positioning of the quadrants reflects the isotype control. Representative plots from 4 individual mice per group are shown. (b) $CD11c^{hi}$MHC class II^{hi}7-$AAD^{-}CD4^{-}CD45^{+}$ DCs were FACS-sorted from pooled cell preparations of colon, MLN and spleen of colitic BALB/c $Rag2^{-/-}$ mice (n = 6). *Cdh1* (*E-Cadherin*) gene expression was assayed in triplicate by qPCR and each sample was normalized relative to HPRT expression. Mean ± S.E.M expression from the triplicate samples is shown. Representative data from 1 of 2 experiments are shown, a further experiment gave similar results.

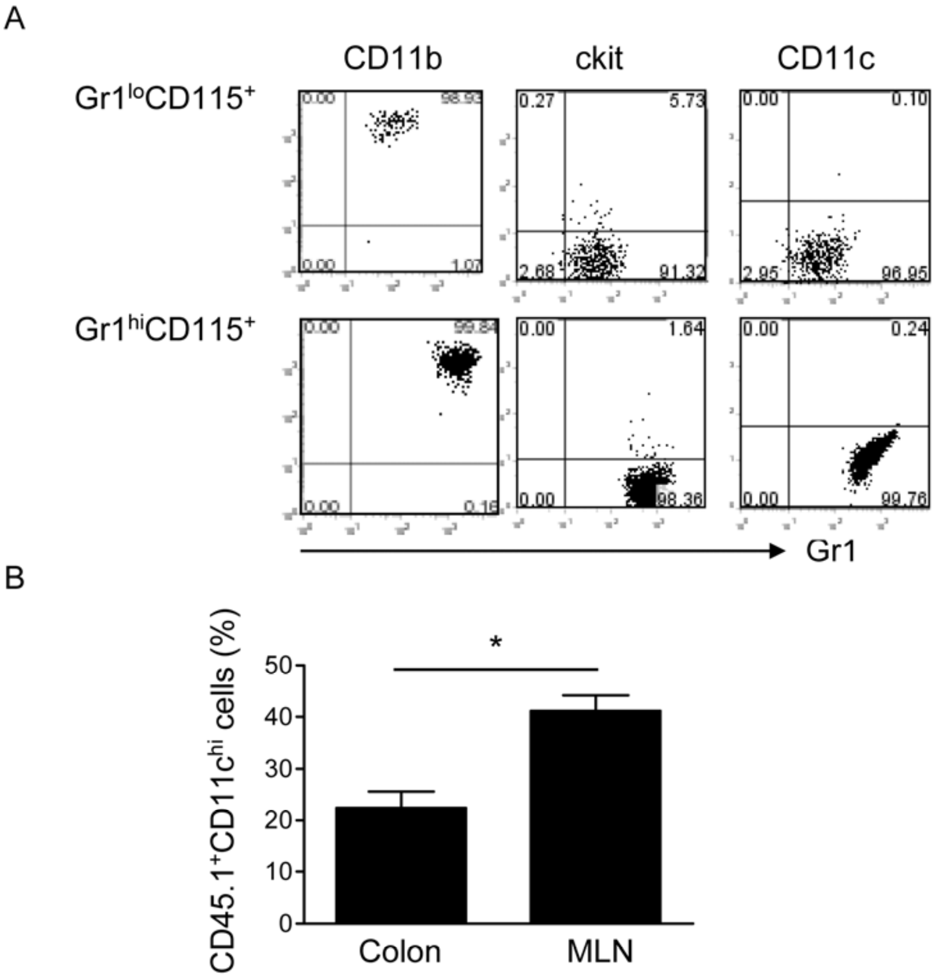

Figure S3. Related to figure 3. Characterisation of $Gr1^+$ and $Gr1^-$ monocytes

(a) Phenotypic characterisation of FACS-sorted $Gr1^+$ and $Gr1^-$ $CD115^+$ monocytes harvested from pooled blood and BM from congenic CD45.1 mice (n = 8). Representative staining of CD11b, ckit and CD11c. Positioning of the quadrants reflects the isotype controls. (b) Mean frequency of $CD45.1^+$ cells expressing $CD11c^{hi}$ derived from $Gr1^+$ monocytes (n = 3). Error bars represent s.d. Representative data from 1 of 2 independent experiments.

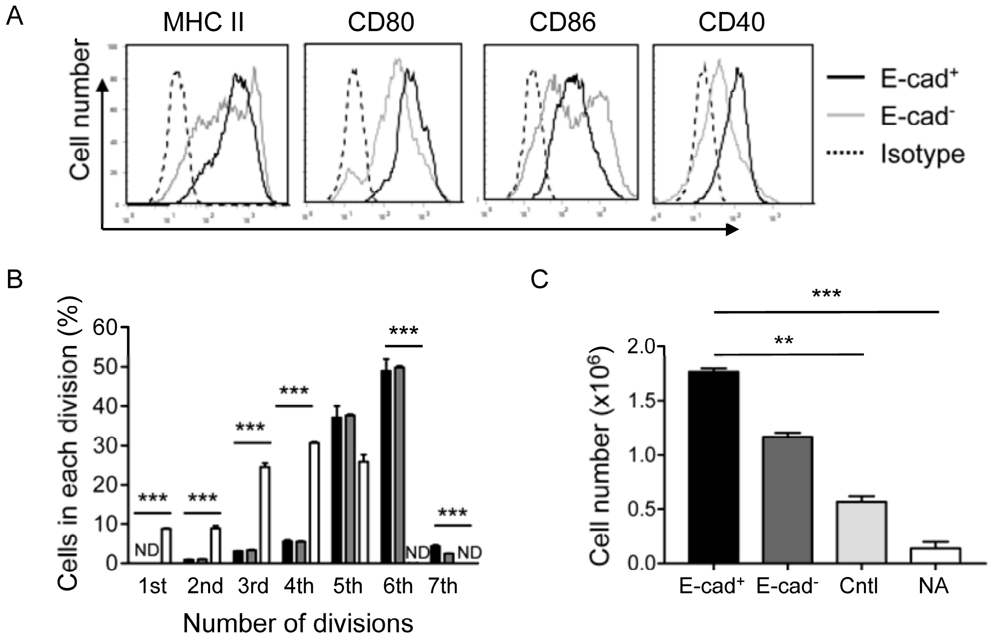

Figure S4. Related to figure 5. MLN E-cadherin^{+} CD11c^{hi} cells from colitic mice display characteristics associated with DCs

(a) Representative staining of MHC class II, CD80, CD86 and CD40 by E-cadherin^{+} and E-cadherin^{-} CD11c^{hi} cells from the MLN of colitic Rag2$^{-/-}$ mice (n = 4). Black solid lines, E-cadherin^{+} DCs; grey solid line, E-cadherin^{-} DCs; and dashed lines, isotype control antibodies. (b-c) 2 x 10^5 CFSE-labelled CD4^{+} T cells from DO11.10 SCID mice were cultured in triplicate with 3 x 10^4 E-cadherin^{+} or E-cadherin^{-} CD11c^{hi}CD103^{-}7-AAD^{-}CD4^{-} cells, or myeloid MHC class II^{+}CD11c^{-}F4/80^{+} cells, taken from the MLN of colitic BALB/c Rag2$^{-/-}$ mice in the presence or absence of OVA protein (5µg/ml). At day 4 of culture, the cells were counted, and T cell CFSE dilutions were assessed by FACS analysis. The percentage of CD4^{+} T cells present in each cell division was determined. (b) Mean percentage of CD4^{+} T cells present in each of the CFSE divisions. Black bar, T cell proliferation driven by E-cadherin^{+} DCs; dark grey bar, T cell proliferation driven by E-cadherin^{-} DCs; open bar, T cell

proliferation driven by myeloid MHC class II^{+}CD11c^{-}F4/80^{+} cells (c) Number of cells in E-cadherin^{+} (black bar), E-cadherin^{-} (dark grey bar) or myeloid (light grey bar) DC-T cell cultures containing OVA protein, or in E-cadherin^{+} DC-T cell cultures without OVA protein (open bar). Error bar represents S.E.M of triplicate cultures. Data from 1 of 2 independent experiments are shown.

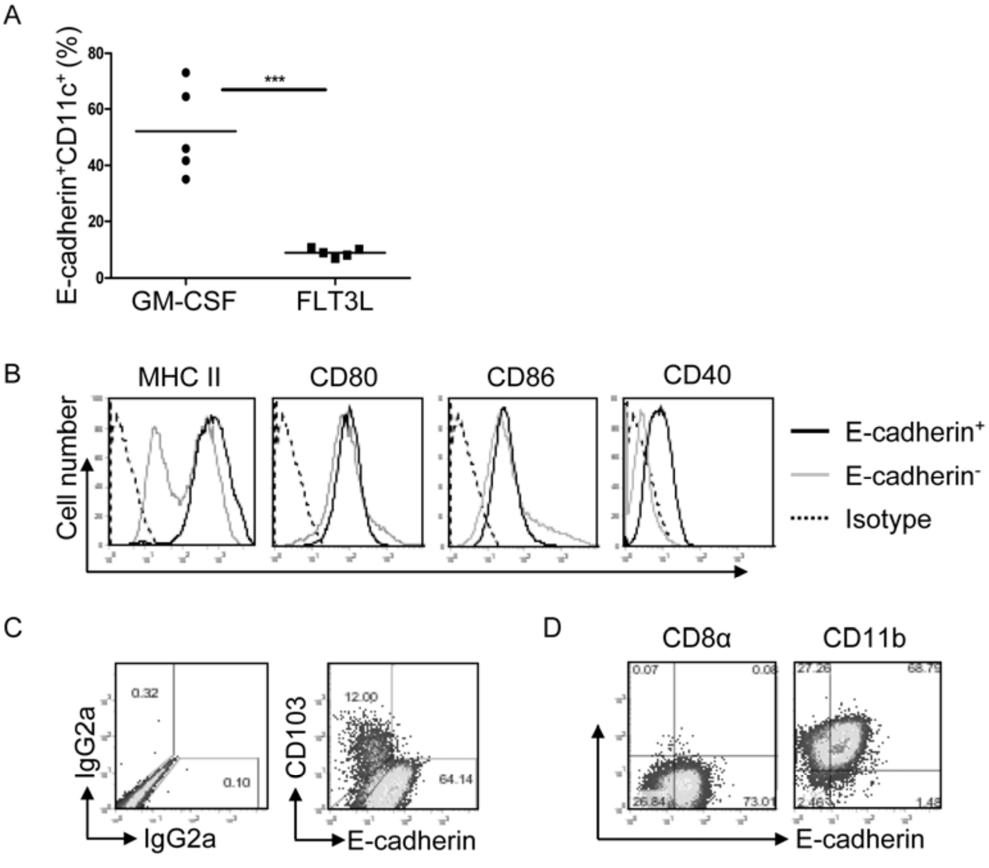

Figure S5. Related to figure 7. Phenotypic characterisation of E-cadherin^{+} BM-DCs

(a-d) BM-DCs were generated in the presence of GM-CSF or Flt3L. At day 8 of culture, cells were harvested, pooled, and the distribution and phenotype of E-cadherin^{+}CD11c^{+} cells were analysed by FACS. (a) Mean percentage of CD11c^{+} cells

expressing E-cadherin following culture in GM-CSF or Flt3L. (b) Expression of MHC class II and co-stimulatory molecules CD80, CD86 and CD40 on E-cadherin^{+} and E-cadherin^{-} CD11c^{+} BM-DCs cultured in GM-CSF. Black solid lines, E-cadherin^{+} BM-DCs; grey solid line, E-cadherin^{-} BM-DCs; and dashed lines, isotype control antibodies. (c) Representative staining of E-cadherin, CD103 and the two relevant isotype control antibodies gated on CD11c^{+} BM-DCs cultured in GM-CSF. (d). Representative staining of E-cadherin, CD8α and CD11b gated on CD11c^{+} BM-DCs cultured in GM-CSF. Positioning of the quadrants reflects isotype controls. In all cases, representative plots from more than 3 independent experiments are shown.
